# Hepatocyte nuclear factors as possible C-reactive protein transcriptional inducer in the liver and white adipose tissue of rats with experimental chronic renal failure

**DOI:** 10.1007/s11010-018-3268-1

**Published:** 2018-01-12

**Authors:** Elzbieta Sucajtys-Szulc, Alicja Debska-Slizien, Boleslaw Rutkowski, Ryszard Milczarek, Iwona Pelikant-Malecka, Tomasz Sledzinski, Julian Swierczynski, Marek Szolkiewicz

**Affiliations:** 1 Department of Nephrology, Transplantology, and Internal Medicine, Medical University of Gdansk, Dębinki 7, 80-211 Gdansk, Poland; 2 Department of Pharmaceutical Biochemistry, Medical University of Gdansk, Dębinki 1, 80-211 Gdansk, Poland; 3 Department of Biochemistry, Medical University of Gdansk, Debinki 1, 80-211 Gdansk, Poland; 4 State School of Higher Vocational Education in Koszalin, Lesna 1, 75-582 Koszalin, Poland; 5 Department of Medical Laboratory Diagnostics, Central Bank of Frozen Tissues and Genetic Specimens, Medical University of Gdansk, BBMRI.PL, Debinki 7, 80-211 Gdansk, Poland

**Keywords:** CRP, Il-6, HNF1, HNF4, LPS, CRF

## Abstract

Inflammation related to chronic kidney disease (CKD) is an important clinical problem. We recently determined that hepatocyte nuclear factor 1α (HNF1α) was upregulated in the livers of chronic renal failure (CRF) rats—experimental model of CKD. Considering that the promoter region of gene encoding C-reactive protein (CRP) contains binding sites for HNF1α and that the loss-of-function mutation in the *Hnfs1*α leads to significant reduction in circulating CRP levels, we hypothesized that HNF1α can activate the *Crp* in CRF rats. Here, we found coordinated upregulation of genes encoding CRP, interleukin-6 (IL-6), HNF1α, and HNF4α in the livers and white adipose tissue (WAT) of CRF rats, as compared to the pair-fed and control animals. This was accompanied by elevated serum levels of CRP and IL-6. CRP and HNFs’ mRNA levels correlated positively with CRP and HNFs’ protein levels in the liver and WAT. Similar upregulation of the *Crp, Il-6*, and *Hnfs* in the liver and WAT and increased serum CRP and IL-6 concentrations were found in lipopolysaccharide (LPS)-induced systemic inflammation in rats. Moreover, silencing *HNF1*α in HepG2 cells by small interfering RNA led to decrease in CRP mRNA levels. Our results suggests that (a) HNFs act in concert with IL-6 in the upregulation of CRP production by the liver and WAT, leading to an increase in circulating CRP concentration in CRF rats and (b) CRF-related inflammation plays an important role in the upregulation of genes that encode HNFs and CRP in the liver and WAT of CRF rats.

**Electronic supplementary material:**

The online version of this article (10.1007/s11010-018-3268-1) contains supplementary material, which is available to authorized users.

## Introduction

Chronic kidney disease (CKD) is a complex disorder affecting multiple human organs and systems. The worsening of kidney function and the accumulation of waste products lead to diverse metabolic, and subsequently clinical, disturbances linked to high morbidity and mortality in CKD patients [1, 2]. Persistent low-grade inflammation is a basic feature of CKD patients. It promotes damage to kidneys and initiates and mediates destructive processes in many organs, particularly in the cardiovascular system, leading to high morbidity and mortality rates [3, 4].

Several biomarkers have been introduced to diagnose and monitor CKD-related inflammatory states [5, 6]. C-reactive protein (CRP) and interleukin-6 (IL-6) have been shown to be the most potent [7]. Many reports have confirmed that serum CRP and IL-6 concentrations are enhanced in CKD patients and are inversely correlated with glomerular filtration rate (eGFR) [8–10]. Panichi et al. [11] showed that IL-6 is a stronger predictor of total and cardiovascular mortality than CRP; however, CRP is the most commonly used clinical marker of inflammation. Moreover, some data suggest that CRP is directly involved in the pathogenesis of coronary heart diseases [12]: it participates in atherogenesis via modulating the expression of genes encoding vascular cell adhesion molecule 1 (VCAM-1), intracellular adhesion molecule 1(ICAM-1), selectin, and monocyte chemotactic protein (MCP-1) in endothelial cells [13–15]. CRP affects basal and stimulated endothelial NO biosynthesis by downregulating the gene encoding endothelial NO synthase (eNOS) [16]. Furthermore, CRP induces upregulation of angiotensin type-1 receptor (AT_1_-R) in vascular smooth muscle cells [17] and plasminogen activator inhibitor-1 (PAI-1) in endothelial cells [18]. Emerging evidence suggests that elevated circulating CRP concentration has become an independent predictor of coronary heart disease [19].

CRP is synthesized mainly by the liver in response to proinflammatory cytokines, particularly IL-6 [14, 20] derived from activated leukocytes [21, 22], adipose tissue [23–25], and in part from the liver [26]. The gene encoding CRP is also expressed in rat [27] and human adipose tissue [28].

Hepatocyte nuclear factor-1α (HNF1α, also known as TCF1 transcription factor-1) is involved in the transcriptional regulation of a large number of hepatic genes, including genes encoding acute-phase proteins and proteins engaged in lipid metabolism [29]. Recently, we have found upregulation of genes encoding HNF1α and HNF4α in the livers of rats with experimentally induced chronic renal failure (CRF) [30]. Given that (a) the promoter region of gene encoding human CRP contains two distinct binding sites, which bind HNF1α and activate the gene encoding CRP [31], (b) the rat *Crp* contains four potential binding sites for HNF1α (TRANSFAC database), and (c) polymorphisms of the *HNF1α* in human affects serum C-reactive protein concentration [32–39], we hypothesize that the elevated level of HNF1α, alongside IL-6, may play a crucial role in upregulating the gene encoding CRP in the liver and white adipose tissue (WAT) of CRF rats. In line with the data reported previously, HNF4α is an upstream transcription factor that activates HNF1α [40]. It is thus very likely that the reciprocal relationship between HNF1α and HNF4α plays a role in the regulation of the expression of genes encoding CRP in experimental CRF.

In this paper, we examine (a) whether HNFs can influence *Crp* expression in the livers and WAT of rats with experimental CRF and (b) whether changes in *Hnfs*’ and *Crp* expression can be associated with inflammation not related with CRF. Moreover, to assess the direct impact of HNF1α on CRP synthesis, we measured the effect of small interfering RNA on *HNF1*α and subsequently on *CRP* expression in HepG2 cells.

Our results suggest that the upregulation of genes encoding HNFs and IL-6 in the liver and WAT of CRF rats is closely associated with the upregulation of CRP. Furthermore, we found that silencing *HNF1*α expression in HepG2 cells RNA led to decrease in CRP mRNA levels. These results suggest that HNFs can act in concert with IL-6 in the upregulation of CRP production by the liver and WAT, leading to an increase in circulating CRP concentration in experimental CRF rats. Moreover, the results obtained with rats treated with lipopolysaccharide (LPS) suggest that CRF-related inflammation plays an important role in upregulating genes that encode HNFs in the liver and WAT of CRF rats.

## Materials and methods

### Animals

The procedures were conducted according to our institutional guidelines for the care and use of laboratory animals.

#### CRF rats

The study was performed using 10-week-old male Wistar rats weighing approximately 250 g at the beginning of the experiment. There were ten animals in each studied group (i.e., CRF, pair-fed, control-sham-operated). CRF was induced by subtotal (5/6) nephrectomy using a dorsal incision [41]. Sham-operated animals served as the control. All animals were kept in individual wire-mesh cages, and CRF and sham-operated rats were allowed free access to tap water and a commercial diet that has been previously described [42]. Pair-fed rats received daily the amount of food corresponding to that consumed by CRF animals and they were allowed free access to tap water. Air temperature in the animal room was set at 22 °C and the lighting schedule was controlled (12-h light/dark cycles). Six weeks after induction of CRF, blood samples were collected from abdominal aorta under thiopental anesthesia and serum was obtained after centrifugation at 1500×*g* for 10 min. The rats were euthanized (between 8.00 and 10.00 a.m). After collection, pieces (~ 0.5 g) of the liver and epididymal WAT were rapidly frozen in liquid nitrogen and stored at − 80 °C until the expression of the studied genes was determined.

#### Clofibrate treatment

Five weeks after the induction of CRF, the rats were given clofibrate (250 mg/kg of body weight for seven successive days) as described previously [43].

#### Lipopolysaccharide (LPS) treatment

Male Wistar rats weighing ~ 250 g at the beginning of the study were used in experiments with LPS. There were ten animals in each group (LPS-treated and saline-treated). Persistent inflammation was induced in LPS-treated rats by implanting a subcutaneous slow-release ALZET osmotic pump (Model 2ML4; Durect Corporation, Cupertino, CA) to infuse 1 mg kg^− 1^ day^− 1^ of LPS (*E. coli* O55:B5; Sigma, Missouri, USA) for 4 weeks. NaCl (0.9%) infusion was used in the saline-treated group. The animals were allowed free access to chow and water with a 12-h light/dark cycle. Rats were anesthetized with ketamine and xylazine after 28 days treatment with LPS. Blood samples were collected from an abdominal aorta and serum was obtained after centrifugation at 1500×*g* for 10 min. Following collection, pieces (~ 0.5 g) of the liver and epididymal WAT were rapidly frozen in liquid nitrogen, and then stored at − 80 °C until the expression of the studied genes was determined.

#### Cell culture

HepG2 cells, a human hepatocellular carcinoma cell line, were obtained from ATCC (ATCC; Manassas, VA). Cells were maintained in standard Minimum Essential Eagle’s Medium (MEM; Sigma) with the addition of 2 mM glutamine, 1% non essential amino acids, 10% fetal bovine serum, penicillin (100 IU per mL), and streptomycin (100 µg per mL). Two days before small interfering RNA (siRNA) transfection, HepG2 cells were passaged in 6-well plates at 10 × 10^− 4^ cells per well. Then the cells were cultured at 37 °C and grown to approximately 70% confluence.

### Small interfering RNA (siRNA) transfection

Two different sequences of siRNA targeting HNF-1α were used: (a) Hs-TCF1-2, No SI00011620, and (b) Hs-TCF1-5, No SI03095015. AllStars Negative Control, No 1027280 was used as negative control (siRNA NC). All siRNAs were obtained from Qiagen (Crawley, UK). HepG2 cells treated by lipofectamine were used as controls (CON). HepG2 cells were transfected with siRNA at concentrations of 10 nM (except for dose—effect studies, in which different concentrations between 0 and 50 nM were examined), using 0.1% (v/v) Lipofectamine RNAiMAX (Invitrogen, Paisley, UK), as described in the manufacturer’s protocol. Transfection reactions were performed in serum-free OptiMEM (Invitrogen, Paisley, UK). Cells were harvested after 48 h and used for total RNA or protein extraction (see below).

### RNA isolation

Total liver and WAT RNA was extracted from the frozen tissue using the guanidinium isothiocyanate—phenol/chloroform method [44]. GenElute™ Mammalian Total RNA Miniprep Kit (Sigma) was used for isolation of total RNA from HepG2 cells. The obtained RNA concentration was determined from the absorbance at 260 nm; all samples had a 260/280 nm absorbance ratio of about 2.0.

### cDNA synthesis

First-strand cDNA was synthesized from 1 µg of total RNA (RevertAid First Strand cDNA Synthesis Kit, Thermo Fisher Scientific, USA). Prior to amplification of cDNA, each RNA sample was treated with RNase-free DNase I (Thermo Fisher Scientific, USA) at 37 °C for 30 min.

### Determination of mRNA levels by RT-PCR

Rat CRP, IL-6, HNF1α, HNF4*α*, β-actin, and TBP (TATA-box binding protein) mRNA levels were quantified by RT-PCR using a Chromo4 Real-Time Detection System (Bio-Rad Laboratories, USA). Primers were designed with Sequence Analysis software package (Informagen, Newington, USA) from gene sequences obtained from the Ensembl Genome Browser (http://www.ensembl.org). The rat sequences of primer pairs (sense and antisense) used in this study are presented in Table A in the Supplementary Appendix. Primers for human: (a) HNF1α (qHsaCED0001918), (b) CRP (qHsaCED0021979), β-actin (qHsaCED0036269), and (c) TBP (qHsaCID0007122) assayed in HepG2 cells, were obtained from Bio-Rad Laboratories, Inc, USA. Real-time PCR amplification was performed in 20 μL volumes using iQ SYBR Green Supermix (Bio-Rad Laboratories, Hercules, CA). Each reaction contained cDNA and 0.3 μM of each primer. Control reactions, with omission of the RT step or with no template cDNA added, were performed with each assay. All samples were run in triplicate. To compensate for variations in the amount of added RNA and in the efficiency of the reverse transcription, β-actin and TBP mRNA levels were quantified in the corresponding samples and the results were normalized to these values. It should be noted that results obtained with β-actin and TBP (as internal standards) were similar. The relative quantities of transcripts were calculated using the 2^− ΔΔCT^ formula [45]. The results are expressed in arbitrary units, with one unit representing the mean mRNA level determined in a control group. Amplification of specific transcripts was further confirmed by obtaining the melting curve profiles and subjecting the amplification products to agarose gel electrophoresis.

### Western blot analysis of CRP, HNF1α, HNF4α, and β-actin

Frozen liver and WAT samples were homogenized in a buffer containing 10 mM Tris–HCl (pH 7.8), 2% SDS, 10 mM DTT, and proteinase inhibitors (Sigma) and centrifuged at 15,000×*g* for 20 min at 20 °C. Supernatants were collected and the protein concentration was determined by Bradford assay. Tissue lysates containing 20 μg (liver) or 60 μg (WAT) of total protein were separated by 10% SDS–PAGE and electroblotted onto Immobilon Transfer Membrane (Millipore). The following antibodies were used: monoclonal antibody against CRP (sc-69770, Santa Cruz Biotechnology), monoclonal antibody against HNF-1 (sc-393925, Santa Cruz Biotechnology), polyclonal antibody against HNF-4 (sc-8987, Santa Cruz Biotechnology), and polyclonal antibody against Actin (sc-7210, Santa Cruz Biotechnology). HRP-conjugated secondary antibodies (sc-2030 and sc-2004) were obtained from Santa Cruz Biotechnology and the HAF019 from R&D Systems. Immunodetection was accomplished with enhanced chemiluminescence using western blotting Luminol Reagent (sc-2048, Santa Cruz Biotechnology).

Harvested HepG2 cells were suspended in 250 mM sucrose, 10 mM Tris–HCl (pH 7.8), 2 mM EDTA, and centrifuged 600×*g* for 10 min at 4 °C. Obtained pellet (containing crude nuclear fraction) was suspended in a RIPA buffer (150 mM NaCl, 1% NP40, 0.5% deoxycholate, 0.1% SDS, 50 mM Tris pH 8.0, complete protease inhibitor cocktail), homogenized, and centrifuged 15,000×*g* for 20 min at 4 °C. Supernatants (nuclear lysate) were collected and protein concentration was determined with Bradford assay. The samples of nuclear lysates, containing 30 μg of total protein, were separated by 10% SDS–PAGE and electroblotted onto Immobilon® Transfer Membrane (Millipore). The following antibodies were used: monoclonal antibody against HNF-1 (sc-393925, Santa Cruz Biotechnology) and polyclonal antibody against proliferating cell nuclear antigen (PCNA) (sc-7907, Santa Cruz Biotechnology).

### Determination of serum CRP and IL-6 concentration

Commercially available ELISA kits were used to estimate IL-6 (R&D Systems, Minneapolis, USA) and CRP (BioVendor – Laboratorni Medicina, Brno, Czech Republic) serum concentrations.

### Serum creatinine and blood urea nitrogen (BUN) concentration

Serum creatinine and BUN concentrations were determined using a Hitachi 704 autoanalyzer.

### Database sequence analysis

The putative HNF1α binding sites of rat *Crp* were sought in TRANSFAC database (BIOBASE, Beverly, MA). The sequence covering 2000 bp upstream and 300 bp downstream the transcription start site of rat *Crp* was analyzed.

### Statistics

The statistical significance of differences between groups was assessed by one-way analysis of variance (ANOVA) followed by Student’s *t* test and one-way analysis of variance (ANOVA), followed by Tukey’s post hoc test. The Sigma Stat software (SyStat) was used. The results are presented as means ± SDs. Differences between groups were considered significant when *p* < 0.05. The relations between two variables were calculated using the Pearson’s correlation.

## Results

To validate CRF experimental model, first we determined serum concentrations of creatinine and BUN, the most commonly used markers of renal function. Serum creatinine and BUN concentrations found in CRF rats were a few times higher than those in control and pair-fed animals (Table 1). Simultaneously, the serum concentrations of CRP were significantly higher in CRF rats when compared with control and pair-fed animals (Table 1). Moreover, circulating CRP concentration positively correlated with serum concentration of creatinine (*r* = 0.92, *p* < 0.001) and BUN (*r* = 0.93, *p* < 0.001). Circulating IL-6 concentration was also significantly increased in CRF rats as compared with control and pair-fed animals (Table 1). Additionally, strong positive correlations between serum creatinine concentration and serum IL-6 concentrations (*r* = 0.65, *p* < 0.001) were found. The above-presented results suggest that in our experimental CRF model circulating CRP and IL-6 concentrations increased in response to renal insufficiency.

**Table 1:** Serum markers of renal function (creatinine, BUN) and markers of inflammatory state (CRP and IL-6) of control (CON), pair-fed (PF), and chronic renal failure (CRF) rats

Markers	CON	PF	CRF
Creatinine (mg/dL)	0.5 ± 0.1	0.6 ± 0.1	2.4 ± 0.7*
BUN (mg/dL)	18.8 ± 4.4	21.2 ± 3.4	95.3 ± 15.3*
CRP (µg/mL)	179.1 ± 19.7	219.0 ± 41	392.3 ± 42.7*
IL-6 (pg/mL)	131.2 ± 15.1	147.4 ± 32	371.2 ± 54.1*

The data are presented as mean ± SD. Statistics: *p<0.05 versus CON and versus PF; *n* = 10 in each group

**Fig. 1:**
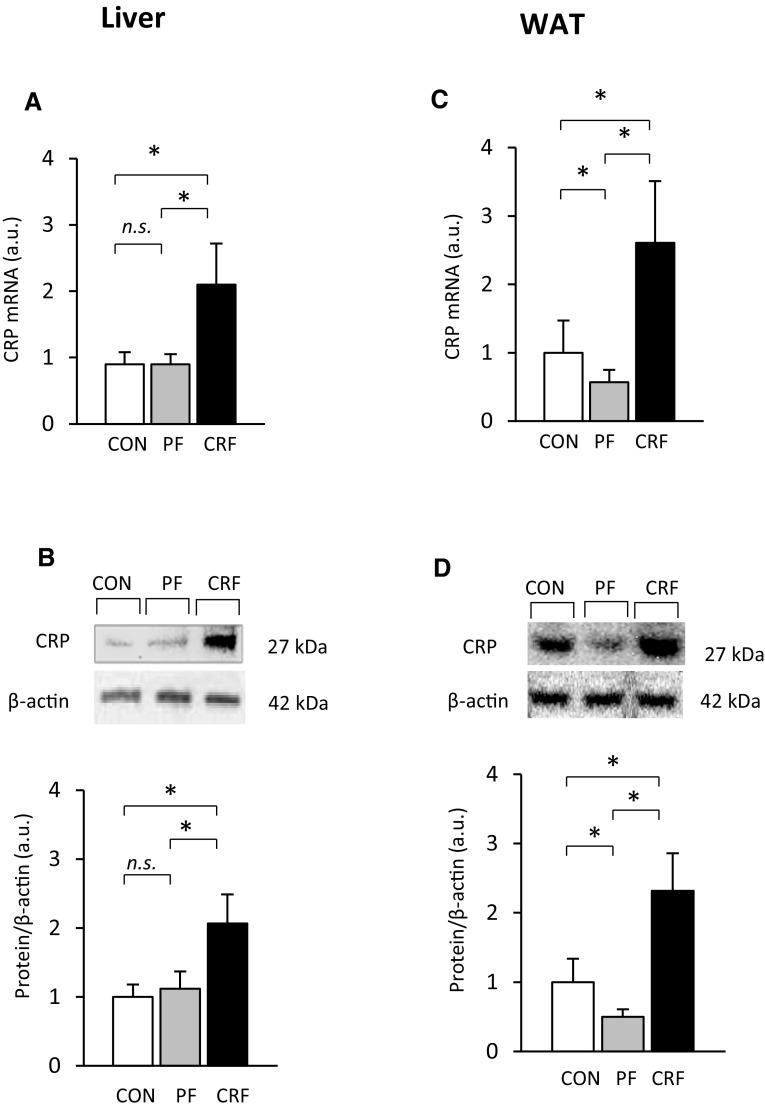
Expression of gene encoding CRP in the liver and WAT of control (CON-empty bar), pair-fed (PF-shaded bar), and chronic renal failure (CRF-filled bar) rats: **a** relative liver CRP mRNA levels; **b** representative western blots (top panel) and densitometric analysis of western blots bands (bottom panel) of liver CRP protein; **c** relative WAT CRP mRNA levels; **d** representative western blots (top panel) and densitometric analysis of western blots bands (bottom panel) of WAT CRP protein. Graphs represent the mean ± SD of results from ten rats. β-actin and TBP mRNA levels were quantified in the corresponding samples and the results regarding CRP mRNA levels were normalized to these values (*a.u.* arbitrary units); for details see “Materials and methods.” β-actin was used as a standard for protein level calculation. Statistics: *p < 0.05, *n.s*. not significant

Liver CRP mRNA levels were approximately twice as high in CRF rats than in the control and pair-fed animals (Fig. 1a). Figure 1b shows, that the abovementioned intergroup differences in CRP mRNA levels were reflected by different levels of CRP protein levels (Fig. 1b, top panel—representative western blots and bottom panel—densitometric analysis of western blots bands). Moreover, CRF was associated with a significant increase in WAT CRP mRNA levels (Fig. 1c); however, a decrease in CRP mRNA levels in the pair-fed group was found. The pattern of changes in WAT CRP mRNA levels was also consistent with the profile of WAT CRP protein levels (Fig. 1d, top panel—representative western blots and bottom panel—densitometric analysis of western blots bands). Overall, the results presented in Fig. 1 indicate that upregulation of gene encoding CRP takes place in the liver and WAT of CRF rats. Additionally, positive correlations between serum creatinine concentration and (a) liver CRP mRNA level (*r* = 0.96, *p* < 0.001) and (b) liver CRP protein amount (*r* = 0.98, *p* < 0.001) were found. Essentially similar relationships between serum creatinine concentrations and (a) WAT CRP mRNA levels (*r* = 0.93, *p* < 0.001), (b) WAT CRP protein levels (*r* = 0.95, *p* < 0.001) were also found.

**Fig. 2:**
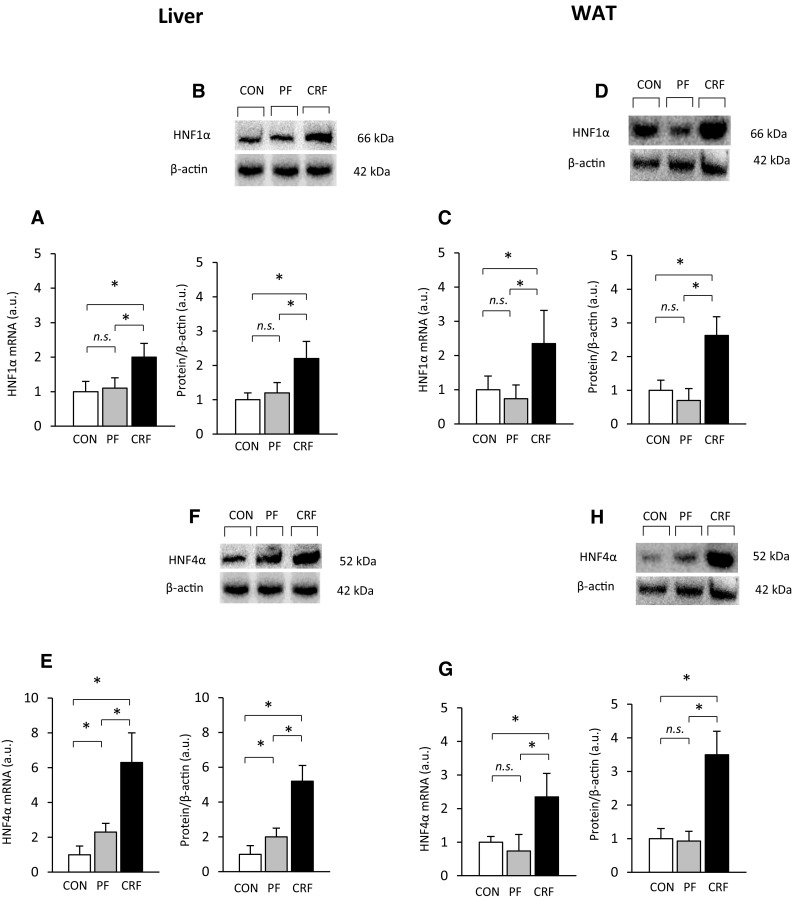
*Hnf1α* and *Hnf4α* expression in control (CON-empty bar), pair-fed (PF-shaded bar), and chronic renal failure (CRF-filled bar) rats: **a** relative liver and **c** WAT HNF1α mRNA levels; **e** relative liver and **g** WAT HNF4α mRNA levels; **b** representative western blot analysis (top panel) and densitometric analysis of western blots bands (bottom panel) of liver HNF1α protein levels; **d** representative western blot analysis (top panel) and densitometric analysis of western blots bands (bottom panel) of WAT HNF1α protein levels; **f** representative western blot analysis (top panel) and densitometric analysis of western blots bands (bottom panel) of liver HNF 4α protein levels; **h** representative western blot analysis (top panel) and densitometric analysis of western blots bands (bottom panel) of WAT HNF4α protein levels. Graphs represent the mean ± SD of results from ten rats. β-actin and TBP mRNA levels were quantified in the corresponding samples and the results regarding HNFs’ mRNA levels were normalized to these values (*a.u.* arbitrary units); for details see “Materials and methods.” β-actin was used as a standard for protein level calculation. Statistics: *p < 0.05, *n.s*. not significant

Upregulation of the gene encoding liver and WAT CRP (data presented above) was closely associated with increases in levels of liver and WAT HNF1α mRNA (Fig. 2a, c, respectively). The pattern of differences in liver and WAT HNF1α mRNA levels was similar to that observed in liver and WAT HNF1α protein levels, as determined by western blot (Fig. 2b, d, respectively; top panel—representative western blots and bottom panel—densitometric analysis of western blots bands). Strong positive correlations between liver HNF1α mRNA level and (a) circulating CRP concentration (*r* = 0.83, *p* < 0.001) and (b) liver CRP mRNA level (*r* = 0.78, *p* < 0.001) were found. Strong positive correlations between levels of liver HNF1α protein and (a) circulating CRP concentration (*r* = 0.95, *p* < 0.001), (b) liver CRP mRNA level (*r* = 0.85, *p* < 0.001), and (c) liver CRP protein levels (*r* = 0.89, *p* < 0.001) were also observed.

Liver and WAT HNF4α mRNA levels were also elevated in CRF rats, compared with the control and pair-fed animals (Fig. 2e, g). The pattern of differences in liver and WAT HNF4α mRNA levels was similar to that observed in liver and WAT HNF4α protein levels (Fig. 2f, h, respectively; top panel—representative western blots and bottom panel—densitometric analysis of western blots bands) of all the control, pair-fed, and CRF rats. Moreover, strong positive correlations between liver HNF4α mRNA and HNF1α mRNA levels (*r* = 0.91, *p* < 0.001), as well as between HNF4α and HNF1α protein levels (*r* = 0.92, *p* < 0.001) were found.

**Fig. 3:**
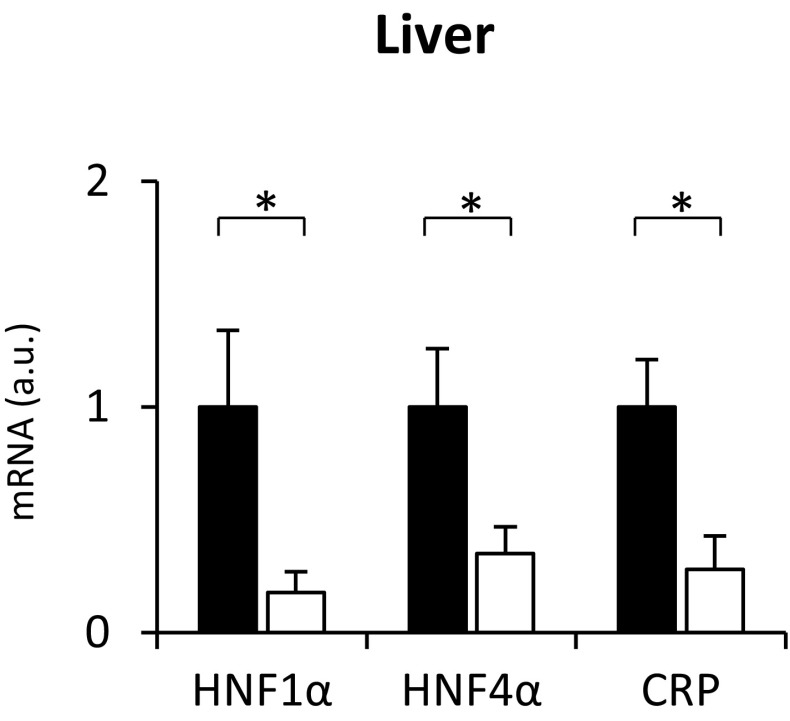
Relative HNF1α, HNF4α, and CRP mRNA levels in the liver of untreated (CRF-filled bar) and clofibrate-treated (CRF+Clofibrate-empty bar) rats with chronic renal failure. Graphs represent the mean ± SD of results from ten rats. β-actin and TBP mRNA levels were quantified in the corresponding samples and the results regarding HNFs’ and CRP mRNA levels were normalized to these values (*a.u.* arbitrary units); for details see “Materials and methods.” Statistics: *p < 0.05

Considering that the promoter region of the gene encoding (a) human CRP contains two distinct regions, which bind HNF1α and activate the gene encoding CRP [31], and (b) rat CRP contains four potential binding sites for HNF1α, it is very likely that upregulation of the *Crp* is a consequence of the increased expression of the *Hnf1α*. Accordingly, treatment with clofibrate, which is capable of decreasing liver mRNA levels of the HNFs [30], coordinately reduces HNF1α, HNF4α, and CRP mRNA levels in the liver of the CRF animals (Fig. 3).

**Fig. 4:**
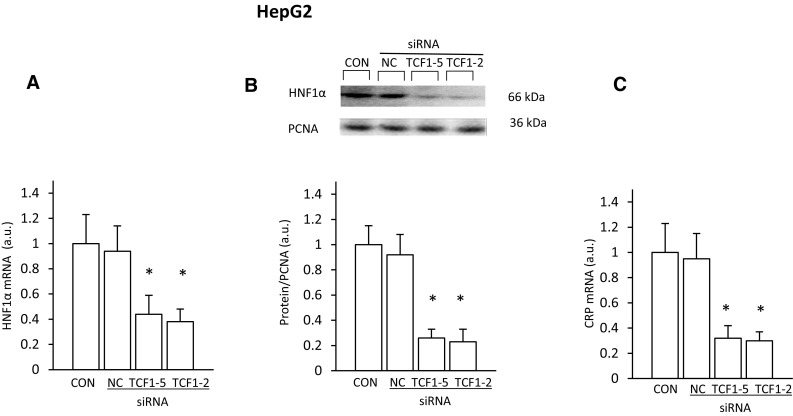
Coordinated inhibition of *HNF1α* and *CRP* expression in HepG2 cells by two different sequences of siRNA targeting *HNF-1α*. **a** HNF1α mRNA level and **b** representative western blot analysis (top panel) and densitometric analysis of western blots bands (bottom panel) of HNF1α protein levels in lipofectamine-treated HepG2 cells (CON), cells transfected with siRNA targeting *HNF-1α* (TCF1-2 or TCF1-5) or negative control (NC). **c** CRP mRNA level in HepG2 cells treated as described above. For other experimental conditions see “Materials and methods.” Graphs represent the mean ± SD of results from 6 plates performed in three different experiments. Statistics: *p < 0.05

To verify if the abovementioned changes in *CRP* expression were truly caused by changes in HNF1α protein levels, we assessed *Hnf1α* deregulation in hepatocellular model (HepG2 cells) by silencing its endogenous expression using small interfering RNA (siRNA). As shown in Fig. 4, the decrease in HNF1α mRNA (Fig. 4a) and HNF1α protein levels (Fig. 4b) by two different siRNAs were associated with the decrease in CRP mRNA level (Fig. 4c). Taken together, the results presented above indicate that HNF1α is involved in the regulation of *CRP* expression in the liver cells (and possibly in WAT), leading to an increase in circulating CRP concentration in experimental CRF rats.

**Fig. 5:**
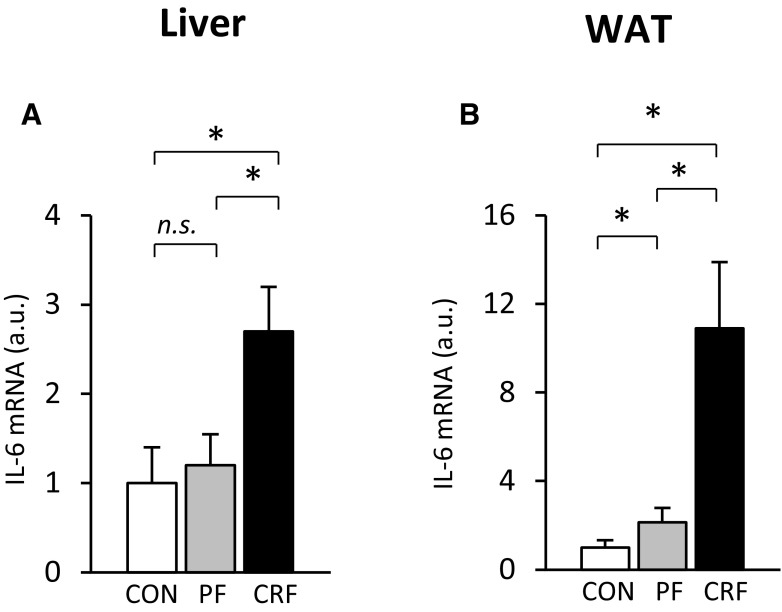
Relative liver (**a**) and WAT (**b**) IL-6 mRNA levels of control (CON-empty bar), pair-fed (PF-shaded bar), and chronic renal failure (CRF-filled bar) rats; Graphs represent the mean ± SD of results from ten rats. β-actin and TBP mRNA levels were quantified in the corresponding samples and the results regarding IL-6 mRNA levels were normalized to these values (*a.u.* arbitrary units); for details see “Materials and methods.” Statistics: *p < 0.05, *n.s*. not significant

It is well known that the gene encoding CRP is upregulated by proinflammatory cytokines, including IL-6 [20], derived partially from adipose [24, 25] and liver [26] tissue. As shown in Table 1, serum IL-6 concentration was significantly increased in CRF rats as compared with control and pair-fed animals. The elevated serum IL-6 concentration was associated with increased liver (Fig. 5a) and WAT (Fig. 5b) IL-6 mRNA levels. Additionally, strong positive correlations between serum creatinine concentration and (a) liver (*r* = 0.62, *p* < 0.001) and WAT (*r* = 0.84, *p* < 0.001) IL-6 mRNA levels and (b) serum IL-6 concentrations (*r* = 0.65, *p* < 0.001) were found.

**Fig. 6:**
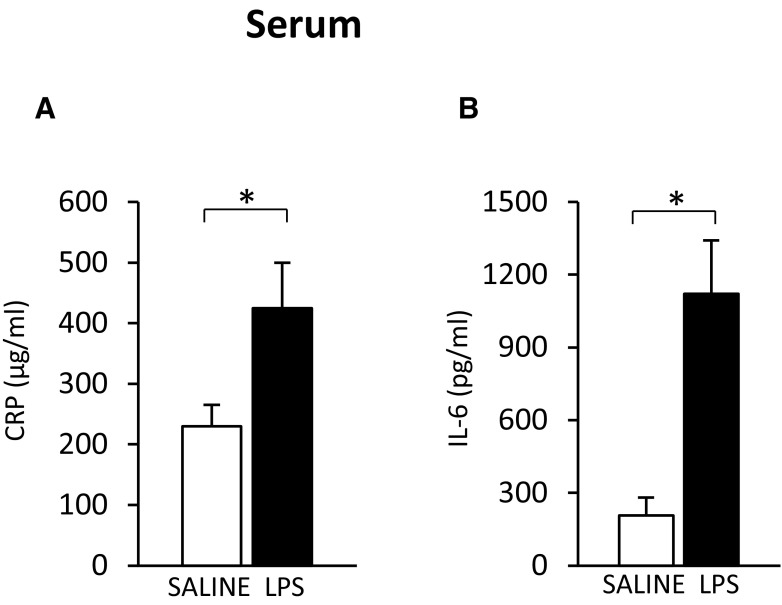
Serum CRP (**a**) and IL-6 (**b**) concentrations in saline-treated (SALINE-empty bar) and lipopolysaccharide-treated (LPS-filled bar) rats. Graphs represent the mean ± SD of results from ten rats. Statistics: *p < 0.05

**Fig. 7:**
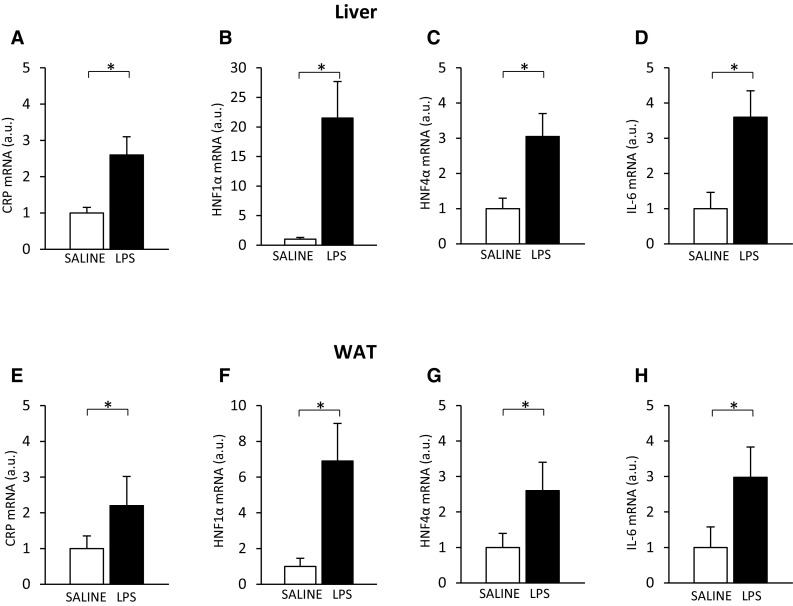
Relative liver CRP (**a**), HNF1α (**b**), HNF4α (**c**), and IL-6 (**d**) mRNA levels and relative WAT CRP (**e**), HNF1α (**f**), HNF4α (**g**), and IL-6 (**h**) mRNA levels in saline-treated (SALINE-empty bar) and lipopolysaccharide-treated (LPS-filled bar) rats. Graphs represent the mean ± SD of results from ten rats. β-actin and TBP mRNA levels were quantified in the corresponding samples and the results regarding HNFs’ and CRP mRNA levels were normalized to these values (*a.u.* -arbitrary units); for details see “Materials and methods.” Statistics: *p < 0.05

These data, along with recently published data [46, 47], suggest that CKD-related inflammation may be responsible for the upregulation of the *Hnf* and *Il-6*, and subsequently the upregulation of the *Crp*. To verify this, we treated healthy rats with LPS, an endotoxin that induces an acute-phase response [48]. LPS administration did not influence serum creatinine (0.6 ± 0.1 vs. 0.5 ± 0.1 mg/dL; n.s.) or BUN (19.8 ± 1.4 vs. 18.6 ± 2.4 mg/dL; n.s.) concentrations, but led to a significant increase in circulating CRP (Fig. 6a) and IL-6 (Fig. 6b) levels. Moreover, the increase in circulating CRP and IL-6 levels observed after LPS treatment was paralleled by an increase in all (a) liver (Fig. 7a) and WAT (Fig. 7e) CRP mRNA levels, (b) liver (Fig. 7b) and WAT (Fig. 7f) HNF1α mRNA levels, (c) liver (Fig. 7c) and WAT (Fig. 7g) HNF4α mRNA levels, and (d) liver (Fig. 7d) and WAT (Fig. 7h) IL-6 mRNA levels. This suggests that the effect of CRF and LPS on the *Crp, Il-6*, and *Hnfs* was essentially similar.

## Discussion

We have shown for the first time that the experimental CRF-related increase in circulating CRP concentration is a result of its overproduction in the liver and WAT. Given that WAT is a much larger organ than the liver (particularly in humans), it can be supposed that the total amount of CRP synthesized in adipose tissue may be quite significant as a source of circulating CRP. This is partly supported by reports indicating that fat mass in obese subjects is strongly correlated with circulating CRP concentration [49].

Moreover, the data presented in this paper suggest that increased liver and WAT CRP biosynthesis may be mediated not only by IL-6 (as is generally accepted), but also by HNF1α. Considering together (a) that the *Crp* is a target of HNF1α [29, 31, 50], (b) the strong positive correlation between *Crp* and *Hnf1α* expression found in the liver and WAT, and (c) the coordinate reduction in the expression of the *Hnf1α* and *Crp* caused by clofibrate (which is believed to reduce *Hnf1α* expression [30]), we propose that HNF1α plays a key role in the upregulation of the *Crp* in CRF rats. Finally, the role of HNF1α in regulation of *Crp* was confirmed by results presented in Fig. 4, which shows that silencing of *Hnf1α* with small interfering RNA (siRNA), led to the decrease in CRP mRNA levels. This is consistent with reports showing that the loss-of-function mutations in the HNF1α-encoding gene associated with maturity-onset diabetes of the young (MODY) lead to a significant reduction in circulating CRP concentrations [51, 52]. Moreover, it has been documented that the polymorphism of *Hnf1α* is associated with the circulating CRP alterations in healthy subjects [53, 54]. All these confirm that *Hnf1α* expression and circulating CRP concentration are closely related to each other, and this phenomenon is observed in various pathophysiological conditions. Thus, it can be supposed that the increased intracellular HNF1α levels found in the liver and WAT of CRF rats may significantly affect CRP synthesis, independently of IL-6, and subsequently increase circulating CRP concentration.

It has previously been shown that *Hnf1α* expression is highly dependent on the intracellular HNF4α level (HNF4α is an upstream HNF1α transcription factor [40]). It is thus very likely that elevated HNF4α levels (caused by the increase in expression of its gene) led to increased HNF1α activity. In turn, through its binding to APRE 1 and APRE 2 sequence of the *Crp* gene, HNF1α may play a crucial role in upregulation of the *Crp* in both the liver and WAT of CRF rats. Given that HNF1α also upregulates the expression of the *Hnf4α* [40], it seems that this reciprocal relationship between HNF1α and HNF4α is highly important in the regulation of *Crp* expression in experimental CRF.

It should be emphasized that HNF1α was initially identified in the liver, but that expression of *Hnf1α* was later found in the pancreas, intestine, and kidney [55]. The data reported here indicate that rat adipose tissue could be added to the list of tissues and organs expressing HNF1α and HNF4α.

Circulating IL-6 is an essential regulator of a number of acute-phase response genes, including the *Crp* [50, 56]. Enhanced liver and adipose tissue CRP synthesis, and subsequently the elevated circulating CRP concentration found in CRF rats, could therefore result from the increased production of proinflammatory cytokines, particularly IL-6 [20]. This study showed a significantly increased circulating level of IL-6 in CRF rats (Table 1), which was associated with higher IL-6 mRNA levels in the liver (Fig. 5a) and WAT (Fig. 5b). Taken together, it seems that the upregulation of the *Il-6* and the *Hnf*s in liver and WAT tissue is closely associated with the upregulation of *Crp*. It seems that IL-6 and the HNFs and act in concert to induce CRP overproduction in the liver and WAT, leading to the high circulating concentrations of CRP observed in experimental CRF rats. Considering the data published by Nishikava et al. [50], the increased levels of serum cytokines (IL-1 and IL-6) present in CRF rats may result in the formation of heteromeric complexes consisting of HNF1α, c-Fos, and STAT3, which may in turn stimulate the expression of the *Crp*. However, further studies are necessary to confirm this suggestion.

The mechanism underlying the upregulation of *Hnf1α* and *Hnf4α* in CRF rats remains elusive. Administration of LPS (1 mg per kg of body mass each 24 h for 28 days) to healthy rats induced upregulation of the liver and WAT *Hnfs*, which was associated with a significant increase in *Crp* expression, despite the fact that renal function did not change. These data suggest that the CRF-related inflammation itself may play an important role in upregulation of the *Hnf*s, and subsequently in the overexpression of *Crp*. It is worth noting that quite different results were presented by Wang et al. [57], who reported a marked decrease in liver HNF4α and HNF1α protein levels in rats following administration of LPS. They suggested that the decrease in HNF4α is primarily the result of the protein post-transcriptional degradation, since hepatic levels of HNF4α mRNA did not change. This discrepancy is likely due to the fact that Wang et al. [57] tested a short-term effect of the acute-phase response induced by a very high dose of LPS (the rats were euthanized only 3 h after administration of a single, very high LPS dose of 5 or 15 mg/kg body mass). They noted that the decrease in the level of HNF4α protein was more pronounced in the rats that received the higher LPS dose. We tested a long-term effect of persistent inflammation induced by a significantly lower LPS dose (with the rats being euthanized after 28 days of continuous subcutaneous administration of the low LPS dose of 1 mg/kg body mass). These results cannot be directly compared with each other, although the long-lasting inflammation induced by LPS seen in our experimental model seems to more accurately resemble the CRF-related inflammatory state.

In conclusion, the results presented here indicate that, in rats with experimental CRF, the liver and WAT *Crp, Il-6, Hnf1α*, and *Hnf4α* are overexpressed. One of the effects of this coordinated activity is an increase in circulating CRP levels. Moreover, in vitro studies indicate that silencing of *Hnf1α* expression by siRNA led to the decrease in CRP mRNA level. The data allow us to recognize that HNFs, alongside IL-6, play an important role in the upregulation of *Crp* gene in the liver and WAT of CRF rats, and presumably in CKD patients. Although the molecular mechanism underlying the upregulation of the *Hnfs* in CRF rats remains elusive, it seems that chronic persistent inflammation plays a crucial role in this process.

## Electronic supplementary material

Below is the link to the electronic supplementary material.

Supplementary material 1 (DOCX 13 KB)
