# Supplementary material for: Hepatocyte nuclear factors as possible C-reactive protein transcriptional inducer in the liver and white adipose tissue of rats with experimental chronic renal failure
Source: Mol Cell Biochem. 2018 Jan 12;446(1):11–23. doi: 10.1007/s11010-018-3268-1 (PMC6096500; doi:10.1007/s11010-018-3268-1)
Supplement: Supplementary file 1 — Supplementary material 1 (DOCX 13 KB) [file 11010_2018_3268_MOESM1_ESM.docx]

Table A

Sequences of oligonucleotide primers used in this study.

| **Gene** | **Primer sequence** |
| --- | --- |
| *Crp* | F: 5'- CATCTGTGCCACCTGGGAGTC-3'  R: 5'- AAGCCACCGCCATACGAGTC-3' |
| *Il-6* | F: 5'- TCCTACCCCAACTTCCAATGCTC -3'  R: 5'- TTGGATGGTCTTGGTCCTTAGCC -3' |
|  |  |
| *Hnf1α* | F: 5'- AAGATGACACGGATGACGATGG -3'  R: 5'- GGTTGAGACCCGTAGTGTCC -3' |
| *Hnf4α* | F: 5'- AAATGTGCAGGTGTTGACCA -3'  R: 5'- CACGCTCCTCCTGAAGAATC -3' |
| *β-actin* | F: 5'- TGTCACCAACTGGGACGATA -3'  R: 5'- GGGGTGTTGAAGGTCTCAAA -3' |
| *Tbp* | F: 5'- CACCGTGAATCTTGGCTGTAAAC -3'  R: 5'- ATGATGACTGCAGCAAACCG -3' |
|  |  |
|  |  |
